# Pharmacological Treatments Inhibiting Levodopa-Induced Dyskinesias in MPTP-Lesioned Monkeys: Brain Glutamate Biochemical Correlates

**DOI:** 10.3389/fneur.2014.00144

**Published:** 2014-08-05

**Authors:** Nicolas Morin, Thérèse Di Paolo

**Affiliations:** ^1^Neuroscience Research Unit, Centre de Recherche du CHU de Québec, Quebec City, QC, Canada; ^2^Faculty of Pharmacy, Laval University, Quebec City, QC, Canada

**Keywords:** Parkinson’s disease, l-DOPA-induced dyskinesia, motor complications, glutamate receptor, basal ganglia, direct pathway, indirect pathway, receptor interaction

## Abstract

Anti-glutamatergic drugs can relieve Parkinson’s disease (PD) symptoms and decrease l-3,4-dihydroxyphenylalanine (l-DOPA)-induced dyskinesias (LID). This review reports relevant studies investigating glutamate receptor subtypes in relation to motor complications in PD patients and 1-methyl-4-phenyl-1,2,3,6-tetrahydropyridine (MPTP)-lesioned monkeys. Antagonists of the ionotropic glutamate receptors, such as *N*-methyl-d-aspartate (NMDA) and α-amino-3-hydroxy-5-methyl-4-isoxazolepropionic acid (AMPA) receptors, display antidyskinetic activity in PD patients and animal models such as the MPTP monkey. Metabotropic glutamate 5 (mGlu5) receptor antagonists were shown to reduce the severity of LID in PD patients as well as in already dyskinetic non-human primates and to prevent the development of LID in *de novo* treatments in non-human primates. An increase in striatal post-synaptic NMDA, AMPA, and mGlu5 receptors is documented in PD patients and MPTP monkeys with LID. This increase can be prevented in MPTP monkeys with the addition of a specific glutamate receptor antagonist to the l-DOPA treatment and also with drugs of various pharmacological specificities suggesting multiple receptor interactions. This is yet to be well documented for presynaptic mGlu4 and mGlu2/3 and offers additional new promising avenues.

## Introduction

Parkinson’s disease (PD) is the most common neurodegenerative movement disorder and is likely to increase due to the aging population (1). PD is principally attributed to the death of dopamine (DA) neurons in the substantia nigra, but other neurotransmitters, such as glutamate, are also affected (2). There is no cure for PD but symptomatic treatments are available (3). Restoring lost DA with its precursor, l-3,4-dihydroxyphenylalanine (l-DOPA), remains the most effective treatment (4). However, many patients (≥40%) develop motor complications after 5–10 years of treatment (5). These motor complications include motor fluctuations and abnormal involuntary movements, such as l-DOPA-induced dyskinesias (LID), and contribute to limit the quality of life in PD patients and can be very difficult to manage (6). Motor fluctuations such as “wearing-off” are also common. Wearing-off is defined as a reduced duration of benefit from an individual l-DOPA dose and a recurrence of parkinsonian symptoms before the next normal dose of l-DOPA (7).

No drug is yet available for LID, aside from some benefit with amantadine that has anti-glutamatergic properties (8). Glutamatergic transmission is increased in the basal ganglia in PD (9) and is also believed to be involved in LID (10, 11).

The mechanisms involved in the occurrence of LID are still not fully understood, altered dopaminergic and non-dopaminergic neurotransmission in the basal ganglia are observed in LID (12). A recent strategy is to treat LID with adjunct drugs targeting non-dopaminergic neurotransmitter systems such as glutamate to indirectly modulate basal ganglia DA neurotransmission (13).

Glutamate is involved in many physiological functions through its interactions with ionotropic glutamate (iGlu), ligand-gated channel, and metabotropic G-protein-coupled glutamate (mGlu) receptors. iGlu receptors drugs suppressing glutamate excitatory transmission often create undesirable side effects (14), whereas acting on mGlu receptors could lead to a more subtle and/or circuit-selective modulation of excitatory transmission (15). Pharmacologic characterization of metabotropic glutamate 5 (mGlu5) receptors and its selective negative allosteric modulators (NAMs) show therapeutic potential in animal models of PD (16–18) and efficacy in human PD (19, 20). While mGlu5 receptors regulate l-DOPA-induced motor behavior, the mechanisms involved remains to be fully elucidated (21).

This review focuses on relevant studies investigating glutamate receptor subtypes in the pathophysiology of PD and LID. Brain biochemical correlates of motor complications in PD patients and 1-methyl-4-phenyl-1,2,3,6-tetrahydropyridine (MPTP)-lesioned monkeys are reviewed.

## Glutamate Neurotransmission in the Basal Ganglia and Parkinson’s Disease

Glutamate is the brain’s most abundant excitatory neurotransmitter mediating as much as 70% of synaptic transmission (22). Amantadine reduces LID, it also improves akinesia, rigidity, and tremor (3). The non-selective inhibitor of glutamate transmission (riluzole) was shown to block l-DOPA-induced motor complications in 6-hydroxydopamine (6-OHDA) lesioned rat model of PD (23, 24) and the glial glutamate transporter GLT1 is increased in dyskinetic l-DOPA-treated 6-OHDA rats (25, 26). However, riluzole was not effective in humans to relieve LID (27, 28).

## Levodopa-Induced Dyskinesias and Non-Human Primate Model

l-DOPA-induced dyskinesias are abnormal involuntary movements seen typically at the peak effect of each dose of l-DOPA in PD patients (3). LID can be viewed quantitatively as an excess of movement or qualitatively as a problem in selecting the appropriate motor program or pattern (3). The mechanisms involved in the occurrence of LID are complex and have been investigated in numerous studies using animal models and parkinsonian patients (29). The loss of nigrostriatal DA and the chronic administration of l-DOPA, or DA agonists, are two necessary conditions for their appearance (30). The development of LID in human usually requires daily treatment for 3–5 years in idiopathic PD (31), whereas for parkinsonism induced by the toxin MPTP it occurs after only weeks or months of treatment (32). The same applies to the MPTP-lesioned monkey where l-DOPA is usually administered daily for weeks before LID appear (33, 34). MPTP-lesioned primates respond to DA therapies as idiopathic PD patients (35, 36) and are currently the best model for studying LID (37).

MPTP-lesioned primates are very useful to test potential antidyskinetic and/or anti-parkinsonian pharmacological agents (37). The primates are rendered parkinsonian and then chronically treated with l-DOPA for several weeks or months until they express stable and well-established LID. Then, acute or chronic effects of compounds are tested when co-administered with l-DOPA (17, 18, 38, 39). This model is widely used since it allows rapid testing of new compounds and animals may be used for several studies. This paradigm is useful to find new treatments for advanced parkinsonian patients with already established LID (37).

Another paradigm uses *de novo* animals rendered parkinsonian with MPTP and then treated with l-DOPA alone or in combination with the agent under investigation (37). This latter paradigm allows the study of specific effects of the test compound on the development of LID and to assess if the effects diminish with long-term use, also called “wearing-off” (40–44). Furthermore, it allows to investigate the post-mortem brains of these monkeys the mechanisms associated with the behaviors and relate it to the specific treatments (42, 44–47). This experiment models newly diagnosed parkinsonian patients when l-DOPA treatment is initiated and could be used to test adjunct drugs to l-DOPA to avoid development of LID while having a good anti-parkinsonian effect (37). Docosahexaenoic acid (DHA) and cabergoline were shown to reduce the severity or delay the development of LID in MPTP-lesioned monkey (41, 48).

## Ionotropic Glutamate Receptors and Levodopa-Induced Dyskinesias

Ionotropic glutamate receptors mediate fast excitatory neurotransmission, whereas mGlu receptors mediate slower modulatory neurotransmission. iGlu receptors are classified into *N*-methyl-d-aspartate (NMDA), α-amino-3-hydroxy-5-methyl-4-isoxazolepropionic acid (AMPA), and kainate (KA) receptors (49). An increase in striatal NMDA and AMPA receptor binding levels in PD patients with l-DOPA-induced motor complications (11) and dyskinetic MPTP monkeys was observed (50, 51). Moreover, NMDA and AMPA receptor antagonists block the development of l-DOPA-induced motor complications in 6-OHDA rats (23). The NMDA antagonist, CI-1041 can prevent the development of LID in parkinsonian monkeys (40), and associated brain molecular changes (52). In these monkeys, CI-1041 also prevented the increased of striatal mGlu5 receptor levels (53). Clinical trials show the antidyskinetic profile of amantadine, known to block NMDA receptors (8, 54, 55). Kynurenic acid antagonizes glycine b site of NMDA receptors, AMPA, and KA receptors (56, 57) and inhibits glutamate release (58). RO 61-8048, an inhibitor of kynurenine hydroxylase activity, can increase kynurenic acid levels (59); it acutely reduced dyskinesias in MPTP monkeys with LID (60) and reduced their development in *de novo* treated MPTP monkeys (61). Abundant recent literature focused on the role of NMDA and AMPA receptor subunits in rodent and non-human primate models of PD in LID including the glycine site, NMDA GluN2D subunits, AMPA receptor subunit composition, and NMDA/AMPA receptor ratio (49, 62–66). Nevertheless, iGlu receptors can cause significant adverse effects such as cognitive impairment in many patients (67, 68).

## Metabotropic Glutamate Receptors and Levodopa-Induced Dyskinesias

Metabotropic glutamate receptors are divided into Group I (mGlu1, 5) coupling to Gq and promoting polyphosphoinositide hydrolysis, Group II (mGlu2, 3) and III (mGlu4, 6, 7, 8) coupling to Gi/Go and inhibiting Forskolin-induced increase in cyclic adenosine monophosphate (cAMP) (69). All mGlu receptors are present in the brain basal ganglia except mGlu6 receptor found primarily in the retina (70). The majority (>90%) of Group I mGlu receptor, including mGlu5, are located postsynaptically on the perisynaptic annulus of dendritic spines (71). Presynaptically localized Group II and Group III mGlu receptors are thought to represent the classical inhibitory autoreceptor mechanism suppressing excess glutamate release from presynaptic terminals (72).

The prototypal mGlu5 receptor antagonist, 2-methyl-6-(phenylethynyl)pyridine (MPEP) and a more selective analog 3-[(2-methyl-1,3-thiazol-4-yl)ethynyl]pyridine (MTEP) (73) improve motor performance (74) and show antidyskinetic activity in 6-OHDA rats (75, 76), but not the other Group I mGlu receptor, such as mGlu1 receptor drugs (77, 78). mGlu5 receptor levels were increased in the putamen of dyskinetic compared to non-dyskinetic MPTP monkeys (42) and parkinsonian patients with motor complications (LID or wearing-off) compared to those without motor complications (53). MPEP and MTEP were shown to have antidyskinetic activity in MPTP monkeys (17) and the mGlu5 receptor antagonist mavoglurant (AFQ056) in MPTP monkeys (18) and humans (19). We reported that development of LID over a month of treatment were lower by overall ~70% with addition of MPEP to the l-DOPA treatment in *de novo* MPTP monkeys (44) and this was associated with a normalization of glutamate (46) and DA neurotransmission (47). Similarly, chronic administration of fenobam to drug-naïve monkeys attenuated the development of dyskinesia without compromising the anti-parkinsonian effect of l-DOPA (43).

Group II mGlu receptor agonists have proven effective in animal models of PD (79). A decrease in mGlu2/3 receptor density in dyskinetic compared to non-dyskinetic MPTP-lesioned monkeys was observed (46). In post-mortem brains of parkinsonian patients, changes in mGlu2/3 receptors were only observed in relation to wearing-off (80).

Recently, agonists of Group III receptors have shown robust efficacy in rodent models of PD (70). mGlu4 receptor agonists reduce γ-aminobutyric acid (GABA)ergic transmission at striatopallidal synapse that is overactive in PD (81, 82). In 6-OHDA-lesioned rats, a combined treatment with l-DOPA and the mGlu4 receptor agonist Lu AF21934 reduced the effective dose of l-DOPA and minimizing the development of LID (83).

Metabotropic glutamate 8 receptor is expressed at lower levels than mGlu4 and mGlu7 receptors but widely distributed in the brain; mGlu7 receptor has low affinity for glutamate only becoming active when glutamate levels are high thus serving as a brake for glutamate overstimulation (70). AMN082, an mGlu7 receptor agonist, was shown to reverse motor dysfunction associated with reduced DA activity in rodent models (84). However, the contribution of mGlu7 and mGlu8 receptors in LID is not yet reported.

## Discussion

**Figure 1:**
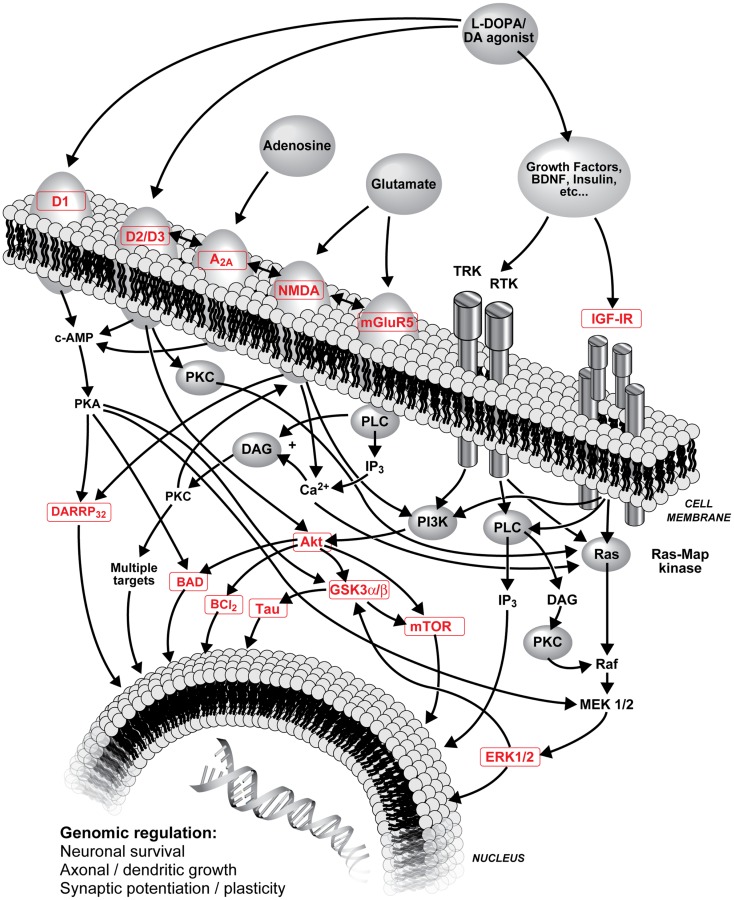
**Schematic representation of l-DOPA (metabolized into DA) and DA agonist treatments on striatal DA neurotransmission, interactions of striatal DA, adenosine, glutamate, and opioids in GABAergic neurons and possible sequence of events leading to LID**. Numerous interactions are known and are not all included; the focus of this figure is on PI3K and MAPK pathways. A_2A_, adenosine A2A receptor; Akt, protein kinase B; BAD, Bcl-2-associated death promoter; Bcl-2, B-cell lymphoma 2; Ca^2+^, calcium ion; cAMP, cyclic adenosine monophosphate; D1/D2/D3, D1/D2/D3 DA receptor; DA, dopamine; DARPP-32, DA and cAMP-regulated phosphoprotein with molecular weight 32; DAG, diacylglycerol; ERK, extracellular-signal-regulated kinase; GSK3, glycogen synthase kinase-3; IGF-IR, type 1 insulin-like growth factor receptor; IP_3_, inositol 1,4,5-trisphosphate; l-DOPA, levodopa (l-3,4-dihydroxyphenylalanine); MEK, mitogen-activated protein kinase; mGlu5, metabotropic glutamate type 5 receptor; mTor, mammalian target of rapamycin; NMDA, *N*-methyl-d-aspartate receptor; PI3K, phosphoinositide 3-kinase; PKA, protein kinase A; PKC, protein kinase C; PLC, phospholipase C; Ras, proto-oncogene protein p21; Raf, proto-oncogene serine/threonine-protein kinase; RTK, receptor tyrosine kinases; Tau, microtubule-associated protein tau; TRK, tropomyosin receptor kinase.

Denervation-induced supersensitivity of DA receptors is generally recognized as a plausible mechanism of LID. Post-mortem studies have shown that DA receptors, particularly D2 subtype, are increased in the striatum of parkinsonian patients (85–87) as well as D1 and D2 receptors in MPTP monkeys (33, 34, 88, 89). However, treatment with l-DOPA can reverse this increase in humans (85, 87) and monkeys (34, 88, 90). LID are clearly more complex than hypersensitivity due to a simple increase in the density of striatal DA receptors (30), hence changes are sought in signaling pathways activated by DA receptors. Various adjunct drugs that can modulate basal ganglia dopaminergic neurotransmission have been shown to treat LID (13, 67, 91–94). Glutamate receptors are reported to interact with numerous neurotransmitters and neuromodulators implicated in the development of LID including dopaminergic neurotransmission (22, 47). Hence, close interactions are described between mGlu5 and NMDA receptors, mGlu5 with D2 receptors, and adenosine A2A receptors (39, 46, 47, 95, 96). Figure 1 shows interactions of striatal DA, adenosine, glutamate and opioids in GABAergic neurons and possible sequence of events leading to LID.

Dopamine receptors are associated with regulation of cAMP– protein kinase A (PKA) through G-protein mediated signaling (97). Downstream from PKA, DA, and cAMP-regulated phosphoprotein with molecular weight 32 (DARPP-32) has important functions in regulating DA receptor signaling and its integration with other signaling modalities (98). Extracellular-signal-regulated-kinase (ERK) is also an important mediator of cAMP signaling involved in responses to DA drugs and might be involved in the development of LID (99–101). Rats with abnormal involuntary movements have abnormally high levels of striatal phospho[Thr34]-DARPP-32 (102). DA receptors also exert their effect through protein kinase B (Akt) and glycogen synthase kinase-3 (GSK3) signaling (97) that might serve to integrate signaling of different receptors such as glutamate. Akt can phosphorylate GSK3β at Ser9 [pGSK3β(Ser9)] and inactivate it (103). GSK3 is a juncture of at least three pathways, mitogen-activated protein kinase (MAPK) (104), phosphoinositide 3-kinase (PI3K) (105), and wingless/integrated-signaling (Wnt) (106). Prolonged stimulation of D2 DA receptors in rodents leads to specific dephosphorylation/inactivation of striatal Akt on Thr308 residue [pAkt(Thr308)], Ser473 [pAkt(Ser473)], remaining unaffected (107). Another downstream protein is mammalian target of rapamycin (mTor) recently reported to be implicated in LID (108).

D1 receptor supersensitive response was shown to result from a switch from normal activation of the PKA cascade to aberrant activation of ERK1/2–MAP kinase in lesioned striata and is suggested to underlie LID (109). Interestingly, in a chronic *de novo* treatment with non-human primates, we observed increases in both striatal pERK1/ERK1 and pERK2/ERK2 ratios of l-DOPA-treated MPTP monkeys whereas MPEP prevented this increase (47). Moreover, there were positive correlations between mean dyskinetic scores and striatal pERK1/ERK1 and pERK2/ERK2 ratios (47). These results suggest that antagonists of mGlu5 receptor can potentially inhibit the excessive striatal activation of nuclear signaling pathways and gene expression that is produced by l-DOPA, which might be related to the interaction with DA receptors.

An association between Akt1 gene and PD was also shown (110). In post-mortem substantia nigra, a large reduction of pAkt(Thr308) and pAkt(Ser473) in PD patients was observed compared to controls (111). l-DOPA-treated MPTP monkeys with LID show elevated pAkt(Ser473) and pGSK3β(Ser9) whereas MPTP monkeys treated with l-DOPA + cabergoline with no LID have lower values (112). In MPTP-lesioned monkeys treated with l-DOPA + CI-1041 that did not develop LID, changes in Akt and GSK3 were modest suggesting implication of other pathways, such as ERK. As in the substantia nigra of parkinsonian patients (111), we observe decreases of striatal pAkt with the MPTP lesion in monkeys (112) whereas in 6-OHDA rats, the lesion did not change or increased phosphorylation of Akt (Ser473 and Thr308) (113). In 6-OHDA rats, pGSK3α and pGSK3β were also unchanged or increased with the lesion (113) while we observed no change or decreases in MPTP monkeys (112). However, both in MPTP-lesioned monkeys (112) and in 6-OHDA rats (113), l-DOPA increased pAkt and pGSK3. Moreover, increase in pAkt(Ser473)/Akt and pGSK3β(Ser9)/GSK3β ratios was observed in the l-DOPA-treated MPTP group, this was prevented with the addition of MPEP and positive correlations were observed between these levels and mean dyskinesia scores (47). This supports a possible involvement of Akt/GSK3β in the mechanisms associated with the development of LID. MPEP might prevent changes in this kinase pathway associated with l-DOPA and could provide new avenues to probe potential novel targets to treat LID.

This mini review focused on glutamate neurotransmission in LID and presented some of its interaction with other neurotransmitter systems showing the complexity of this motor complication and its treatment. Indeed, altered dopaminergic and non-dopaminergic neurotransmission, including also serotonergic, adenosine, cannabinoid, opioid, GABAergic, adrenergic, histaminergic, and cholinergic systems are observed in LID (12, 51). For example, serotoninergic dysfunctions in LID are well documented (114, 115) and serotonin neurotransmission can interact with iGlu (116–118) and mGlu receptors (119).

## Conclusion

Nigrostriatal denervation in PD leads to increased glutamatergic transmission in the basal ganglia; increased glutamate neurotransmission is also observed in LID. These observations suggest that glutamate receptor stimulation is involved in the pathogenesis of l-DOPA-induced motor complications in PD and glutamate receptor subtypes, such as mGlu5 and NMDA receptors, are potential selective targets for treatment of these adverse effects. Recent studies point to changes in activation of DA receptor signaling in LID rather than changes in DA receptor density. Post-mortem brains of dyskinetic MPTP-lesioned monkeys and PD patients treated with anti-glutamatergic drugs and inhibiting LID show multiple brain molecular changes suggesting various receptors interactions. Thus, ionotropic and metabotropic glutamate receptors represent interesting targets to reduce and prevent LID as well as to prevent associated molecular changes beyond their specific receptor target.

## Conflict of Interest Statement

The authors declare that the research was conducted in the absence of any commercial or financial relationships that could be construed as a potential conflict of interest.

## Abbreviations

6-OHDA, 6-hydroxydopamine; Akt, protein kinase B; AMPA, α-amino-3-hydroxy-5-methyl-4-isoxazolepropionic acid; cAMP, cyclic adenosine monophosphate; DA, dopamine; DARPP-32, DA and cAMP-regulated phosphoprotein with molecular weight 32; DHA, docosahexaenoic acid; ERK, extracellular-signal-regulated kinase; GABA, γ-aminobutyric acid; GSK3, glycogen synthase kinase-3; GP, globus pallidus; GPe, external globus pallidus; GPi, internal globus pallidus; iGlu, ionotropic glutamate; KA, kainate; l-DOPA, levodopa (l-3,4-dihydroxyphenylalanine); LID, l-DOPA-induced dyskinesias; MAPK, mitogen-activated protein kinase; mavoglurant, AFQ056; mGlu, metabotropic glutamate; MPEP, 2-methyl-6-(phenylethynyl)pyridine; MPTP, 1-methyl-4-phenyl-1,2,3,6-tetrahydropyridine; MTEP, 3-[(2-methyl-1,3-thiazol-4-yl)ethynyl]pyridine; mTor, mammalian target of rapamycin; NAM, negative allosteric modulator; NMDA, *N*-methyl-d-aspartate; PD, Parkinson’s disease; PI3K, phosphoinositide 3-kinase; PKA, protein kinase A; PP-1, protein phosphatase-1; STN, subthalamic nucleus; Wnt, wingless/integrated-signaling.
